# The functioning and behaviour of biological parents of children diagnosed with attention-deficit/hyperactivity disorder, attending the outpatient department at Weskoppies Hospital, Pretoria

**DOI:** 10.4102/sajpsychiatry.v22i1.836

**Published:** 2016-05-31

**Authors:** Ravindra Sundarlall, Debbie Van der Westhuizen, Lizelle Fletcher

**Affiliations:** 1 Faculty of Health Sciences, University of Pretoria, South Africa

## Abstract

**Background:**

ADHD (attention-deficit/hyperactivity disorder) is gradually being acknowledged as a functionally impairing disorder across the lifespan, underscored by heritability. Nonetheless, lack of ADHD (adult attention-deficit/hyperactivity disorder) data from South Africa is alarming which could be due to either the unawareness of ADHD symptoms or underutilisation of available screening measures. Undiagnosed ADHD may influence family- and working lives unpleasantly. Parenting a child with ADHD may intensify parental stress through functional impairment notwithstanding the diagnosis of ADHD.

**Methods:**

Eighty-one biological parents of children diagnosed with attention-deficit/hyperactivity disorder were screened using self-reporting measurements. ADHD self-report scale (ASRS-V 1.1) identified either positive or negative subgroups; the Weiss functional impairment rating scale (WFIR-S) for functional impairment and the Jerome driving questionnaire (JDQ) for risk-taking behaviour specifically driving.

**Results:**

Of the 39 (48%) parents who experienced impairment in all seven areas of functioning, 23 (59%) screened negative for ADHD, while 16 (41%) screened positive. A significant association was found between parents who screened either positive or negative for ADHD and functional impairment across five of the seven individual categories namely family, work, self-concept, life-skills and social functioning.

**Conclusion:**

This study emphasised the high incidence of functional impairment in parents of ADHD children. Although a substantial number of parents screened negative for ADHD, they still reported impairment in functioning; probably due to undiagnosed ADHD with comorbid psychiatric disorders, and/or parental stress due to the complex behaviour of the child. Parents of children diagnosed with ADHD should be screened for functional impairment followed by referral for psychiatric assessment and parent management training to achieve better clinical outcomes.

## Introduction

This study was motivated by a lack of prevalence data from the South African context on parental functional impairment with regard to parental ADHD and the incapacitating effects of having to raise a child with ADHD.

Research on how raising a child with ADHD affects the functioning of parents, has received little attention.^1^ A Danish study has revealed a causal association between poor child health and parental outcomes, focussing on CADHD (child attention-deficit/hyperactivity disorder) and ADHD.^1^ Empirical studies showed that parents of children with learning or behavioural problems such as those in ADHD, experience higher levels of stress than other parents.^2^

Parental stress developing from parenting an ADHD child is a complex construct involving behavioural, cognitive, and affective components that could manifest in a strained parent-child relationship.^3^ Early identification of potential at-risk parents is necessary so that interventions aimed at reducing the stress in the child-parent systems can be implemented to reduce future behavioural and emotional problems for both parent and child.^1^

Descriptions of ADHD appeared in the psychiatric literature from 1976.^4^ Studies showed that ADHD persists during adolescence and into adulthood in around 67% of individuals either as ADHD or in ‘partial remission’ (having minimal symptoms and/or functional impairments).^4^ Neuropsychological and neuroimaging studies, genetics, persistent and co-morbid psychiatric disorders in adults, response to treatment, and follow up studies of ADHD children to adulthood, support this trend in validating adult ADHD.^5^ In recent international studies, the prevalence of ADHD was in the range of 2%–5%.^4,6^ Persistent forms of ADHD have a higher familial loading than non-persistent types.^7^

There is growing recognition of the importance of ADHD in the parents of children with ADHD, as close to 20% of parents will have ADHD themselves.^4^ Identified genetic associations for heritability in ADHD are close to 76%.^8^ A survey has shown that lower income countries report a lower prevalence (1.9%) than higher income countries (4.2%).^9^

In a recent South African study, Mohammedy et al. studied the persistence of symptoms of CADHD into adulthood.^10^ They found a prevalence rate of 36.4% of ADHD symptoms in parents of ADHD children with an increase in substance abuse, unemployment, depressive and bipolar disorders.

Cheesman explored parenting stress levels in parents raising ADHD children.^11^ The results of this study underscored the immense stress and impairment these families dealt with. An increase in the severity of ADHD symptoms was directly associated with higher levels of family dysfunction, even after controlling for comorbid disorders with lower levels of social support and quality of life.^12^

In addition, professional ignorance of ADHD has far-reaching consequences for ADHD parents, namely poverty, limited education, less professional employment, more frequent changes in employment, poor work performance, higher rates of quitting or fired from jobs. Barkley et al. found elevated rates of speeding citations, suspended licenses and crashes with or without bodily injury in ADHD.^5^

DSM-5 recognises that impairment from the symptoms of ADHD may develop later in life and sometimes appear by early adolescence-by age of 12 years. The reduction in the symptom threshold for adults as compared to children recognises the age-dependent changes where there is clear evidence of functional impairment with or without symptoms of ADHD.^4^ These changes mean that many people who previously met the ‘in partial remission criteria’ will meet full criteria for ADHD under the DSM-V.^13^ Adults often underreport their symptoms, perhaps considered them as part of their personality rather than abnormal.^9^

Parenting an ADHD child with the parent possibly having ADHD is a relatively new and challenging area of research, given the high heritability and comorbidity of ADHD.^12^

We hypothesized that the biological parents of ADHD children would show a similar clinical picture as their child; adult functional impairments and risk taking behaviours could be associated with symptoms of ADHD and/or high stress levels from parenting a child with ADHD.

This study focussed on functional impairment, risk-taking behaviour related to driving, and to screen for negative or positive ADHD (undiagnosed or subclinical-where biological parents did not meet the full diagnostic criteria).^13^

## Method

The protocol was approved by the Faculty of Health Sciences Research Ethics Committee of the University of Pretoria.

A sample of eighty-one parents of previously diagnosed ADHD children was screened (either the biological mother or father) who had neither a self-referral nor a self-report of an ADHD diagnosis before. Screening took place at the Weskoppies Adolescent and Child Psychiatric Out-patient Unit.

Informed consent was obtained from the participants. Three sets of self-reporting questionnaires were handed out to the identified parents for completion. All participants were literate and fluent in English.

The Adult Self-report Scale^14^ (ASRS – V1.1) was used to screen for core symptoms of ADHD. If part A has four or more responses, then it was considered as more predictive of a definitive diagnosis of ADHD. An adaptation of the Weiss Functional Impairment Rating Scale–Self-report (WFIRS – S)^14^ was used to assess functional impairment in the family, work, school, life skills, self-concept, social aspects and risk. The Weiss functional impairment rating scale (WFIRS-S) was scored by the average of the total number of items per category. When defining impairment for clinical purposes, clinicians consider any category with a mean score ≥ 1.5 as impaired.^14^ An adaptation of the Jerome driving questionnaire (JDQ)^14,15^ was used as a self-reporting instrument of which only part B was used for the purposes of the study. The JDQ provided a subjective account of driving style (in terms of levels of alertness, emotional stability, impulsivity and attention) on a scale from zero to ten.^14,15^

The focus of the analysis was a comparison of respondents who screened positive and negative for ADHD, based on the ASRS-V1.1 instrument. Univariate statistics were reported. Due to a large number of missing values, resulting in relatively small sub-sample sizes when the data set was split, nonparametric tests were performed. Fisher’s exact test (reported with the Chi-square test results) were used to assess the relationship between parents who screened positively and negatively for ADHD and each of the functional impairment categories. Mann-Whitney’s test for two independent samples (analogous to an independent sample T-test) was used to evaluate the two parent groups with respect to the four driving style elements (JDQ Part B).

Analysis was conducted using SPSS (Statistical Package for Social Sciences) version 22.^16^

### Results from Questionnaire data analysis

Of the 81 (*N*); parents in this study, 57 (70%) were females. The mean age for males was 42.04 (SD 5.77) and 40.33 (SD 7.61) for females. Just over half of the parents were unemployed.

According to the ASRV self-report scale response parents were classified into two groups, those who screened negative for ADHD (*n* = 63; 77.8%) and those who screened positive for ADHD (*n* = 18; 22.2%). Of the 18 (22.0%) respondents that screened positive for ADHD, 15 (83.3%) were females.

Tables 1–3 summarise the results of impairment in parental functioning as measured by the Weiss impairment rating scale according to parents who scored positive and negative.

**TABLE 1:** Descriptive findings of the most impaired items of functioning within each of the main categories of parental functional impairment (WFIR-S).

WFIR-S Categories	*N*	*n* im-paired (%)	% ADHD Positive	% ADHD Negative	Cron-bach’s *α*	Items per category that were most impaired
Family	75	60 (80)	24	56	0.84	Spouse or partner relationships, family, balancing own needs vs family, losing control
Work	52	22 (42)	21	21	0.82	Work done, performing duties, working to potential, poor performance evaluations, supervisor problems, coming late, keeping a job
School	66	28 (43)	14	29	0.87	Taking notes, completing assignments, inconsistent grades
Life-skills	80	57 (71)	21	50	0.83	Sleeping, managing money, avoiding exercise, doing chores, getting to bed, sex
Self- concept	75	61 (81)	24	57	0.92	Feeling frustrated, discouraged, incompetent, not happy
Social	74	38 (51)	20	31	0.90	Arguments, hobbies, fun with other people
Risk behaviour	71	23 (33)	13	20	0.75	Smoking cigarettes, drinking alcohol, verbally aggressive

**TABLE 2:** Descriptive findings of ADHD subgroups for functional impairment and tests for significance between the two groups.

WFIR-S categories	Functional Impairment	ASRS-V1.1		*n*	Missing in the data	*p*-Fishers exact test

		Positive ADHD *f*_o_ (%)	Negative ADHD *f*_o_ (%)			
Family	Yes	18 (100)	42 (74)	60		**0.016**
	No	0 (0)	15 (26)	15		
**Total**		**18**	**57**	**75**	**6**	
Work	Yes	11(78.6)	11 (29)	22		**0.002**
	No	3 (21.4)	27 (71)	30		
**Total**		**14**	**38**	**52**	**29**	

School	Yes	9 (64.3)	19 (36.5)	28		**0.075**
	No	5 (35.7)	33 (63.5)	38		
**Total**		**14**	**52**	**66**	**15**	

Life	Yes	17 (94.4)	40 (64.5)	57		**0.016**
Skills	No	1 (5.6)	22 (35.5)	23		
**Total**		**18**	**62**	**80**	**1**	

Self-concept	Yes	18 (100)	43 (75.4)	61		**0.032**
	No	0 (0)	14 (24.6)	14		
**Total**		**18**	**57**	**75**	**6**	

Social	Yes	15 (83.3)	23 (41.1)	38		**0.002**
	No	3 (16.7)	33 (58.9)	36		
**Total**		**18**	**56**	**74**	**7**	

Risk behaviour	Yes	9 (50)	14 (26.4)	23		**0.084**
	No	9 (50)	39 (73.6)	48		
**Total**		**18**	**53**	**71**	**10**	

**TABLE 3:** Descriptive findings for parents within the ADHD subgroups and in all seven categories of the WFIR-S (*n* = 39).

Subgroups	Functionally Impaired in all seven categories (WIFR-S)		Gender		Total

			Male *n* (%)	Female *n* (%)	
Screened Negative ADHD	-	yes	9 (23.1)	14 (35.9)	23 (59.0)
	-	no	12	28	40
**Total**			**21**	**42**	**63**

Screened Positive ADHD	-	yes	0	2	2
	-	no	3 (7.7)	13 (33.3)	16 (41.0)
**Total**			**3**	**15**	**18**

Functionally impaired in all seven categories (WIFR-S)	-	yes	12 (31.0)	27 (69.0)	39 (100)
	-	no	12	30	41
**Total**			**24**	**57**	**81**

A significant association was found between parents who screened either positive or negative for ADHD and functional impairment per individual category across five of the seven categories namely family, work, self-concept, life-skills and social functioning. The internal consistency of the WFIRS – S categories and the subgroups was good (Table 1).

This parent sample - including both ADHD subgroups - was more functionally impaired in the self-concept (81%), family (80%) and life-skills (71%) categories than in the work, social, school and risk categories. The categories within the ADHD subgroups showed that functional impairment was more evident in those parents who screened negative for ADHD than those who screened positive for ADHD with reference to categories (Table 1).

Approximately 50% (39) of the 81 parents (positive and negative subgroups) were impaired in all categories of which 30.8% were males (*n* = 12) and 69.2% females (*n* = 27) (see Table 3).

### ADHD symptoms compared with functional impairment categories

The findings of the WIFR-S as per the ADHD subgroups are represented in Table 2. All of the categories in the ADHD positive group had a larger proportion of the impairments compared to the ADHD negative group. The ADHD positive group contributed a larger proportion to each category supporting our hypothesis.

Of the ADHD positive group, all parents (*n* = 18) were 100% impaired in the family and self-concept categories, whereas life-skills impairment fell short by one parent. Almost 90% (*n* = 16) from the positive subgroup were impaired in all seven domains, which accounted for 20% of the total parent sample, shown in Table 3.

Descriptive findings of ADHD subgroups for functional impairment and tests for significance between the two are reported in Table 2. The results from Fisher’s exact test and frequencies demonstrated that statistically significant associations were found for five of the seven functional impairment categories, while school and risk showed moderate association (*p* < 0.1). These findings gave an indication of the extent to which the two ADHD subgroups contributed to impairment in family, work, school, social, life-skills, and self-concept and risk categories.

### Adapted JDQ Driving risk and ADHD self-report symptoms

Of the 39 parent drivers who represented just under 50% with a valid driver’s licence, 6 (15%) were from the positive and 33 (85%) from the negative ADHD subgroups (Table 4). On average, emotional instability and impulsivity were higher in the ADHD positive subgroup than the negative group.

**TABLE 4:** Descriptive findings and tests of significance; Mann-Whitney U for the four driving constructs within the ADHD subgroups (ASRS V1.1).

JDQ driving constructs	ADHD Positive		ADHD Negative		Mann-Whitney *U*	
		
	M	SD	M	SD	*p*-value
1. Level of alertness	2.16	2.01	2.21	2.09	0.835
2. Level of emotional instability	3.50	2.79	2.33	2.12	0.311
3. Level of impulsivity	4.50	3.47	2.42	2.59	0.184
4. Level of inattention	2.95	1.72	3.25	1.77	0.662

M, mean; SD, standard deviation.

Table 4 displays the results of the Mann-Whitney tests which evaluated differences between the two ADHD subgroups. The results in the last column of Table 4 show that the ADHD subgroups do not differ significantly with regards to driving style on any of the four dimensions (level of alertness, emotional liability, impulsivity and inattention). The results confirm that the adapted JDQ driving style assessment, functions independently of psychiatric diagnoses and functional impairment.

### Demographics and ADHD subgroups

The average age of the parents was close to 43 years, which suggests an appropriate adult sample for this study. There was a larger imbalance in their gender groupings. This study had predominance (70%) of female parents, who represented a larger proportion for employment status. There was also a predominance of females in the ADHD subgroups with impairment in functioning. The high unemployment rate could also explain this finding.

Functional impairment was positively associated with age, although the gender composition of the sample attenuated this association with females (Table 2).

### Functional impairment in biological parents of ADHD children

There were significant impairments in the following categories’ of functioning; self-concept, family, life skills, social and work categories when controlling for ADHD positive screened parents (Table 1). These findings indicate that other factors were indeed present in the negatively screened ADHD subgroup, which this study did not test for directly. These findings mirrored the dynamics of a dysfunctional family, such as co-morbid psychiatric disorders and/or parental stress associated with parenting CADHD; not excluding parental ADHD and were highly consistent with the findings of other studies internationally.^4^

The first study in South Africa that highlighted the effect of the ADHD child on the parent and the family was completed by Cheesman.^11^ This suggests a lack of ADHD research in Africa. Internationally, parental outcomes were by Wymbs who reported a higher divorce rate for parents of children with ADHD.^1^

Children with ADHD often demonstrated, oppositional-defiant behaviour and conduct problems resulting in increased levels of parental stress, low parenting self-efficacy, low parental self-esteem and guilt. No studies to date, addressed psychosocial interventions for ADHD and comorbid disorders in preschool-aged children or adults.^12^

Disrupted parental sleep may include poorer daytime functioning, including greater daytime sleepiness, lowered frustration tolerance, impaired attention and memory. Poor sleep in the positive and negative ADHD subgroups could also be an explanation for the non-significant findings of the adapted JDQ variables independent of diagnoses in this study.^14,15^

### ADHD self-report symptoms compared with functional impairment categories

Epidemiological studies tend to find subjects that are often unaware of a disorder but may be aware of impairments. Symptoms of ADHD decline with age at the same time, functional impairment and low socio-economic outcome prevalence rates increase even with a reduced number of symptoms.^17^ Observing this in this study suggests a reduced prevalence of adult ADHD occurs as an underestimation of its true prevalence.

Adults with ADHD have strained relationships with their children who are diagnosed with ADHD and other family members. They are often unable to be emotionally supportive, have poor listening skills, have confounding family activities and are unable to sustain routines-including treatment regimens.^18^

In comparison with the two subgroups in this study, having problems with family, spouse/partner, balancing the needs against, and losing control within the family category were more problematic within the category of the family impairment ( *p* = 0.016) (Table 2).

In the school category of impairment ( *p* = 0.075), taking notes, completing assignments, inefficient completion of work, not working to full potential and inconsistent grades was most problematic in the parent sample (Table 2) Biederman et al. found a statistically significant result regarding these issues.^18^

The problems from school continue into the workplace resulting in high unemployment rates and frequent job changes, which results in less financial resources.^4^ In the work category of impairment ( *p* = 0.002) more females were unemployed, had problems getting work done, performing required duties, working to their potential, being late, poor performance evaluations and keeping a job (Table 2).

Problems with getting into bed, sleeping, avoiding exercise, managing money, keeping up with household chores and sex were most affected in the life skills category of impairment ( *p* = 0.016). In the category of self–concept, an increased tendency ( *p* = 0.032) in low self-esteem, being frustrated, discouraged, not happy with life and incompetent were significant. The limited self-optimism and self–concept indicate decreased quality of life (Table 2). Biederman et al. described an increase in sexual problems that were also reflected in this study which are being recognised as an important quality of life issue.^5^

The most problematic items in the social category of impairment ( *p* = 0.002) are getting into arguments, poor participation in extra-mural activities and inappropriate talking. Due to these handicaps, a number of parents feel isolated, lonely and ashamed about failures, even in the presence of a high IQ.^4^ The risk behaviour category ( *p* = 0.084) is approaching significance on the items that were increased; smoking cigarettes, drinking alcohol and being verbally aggressive (Table 2).

Borland and Heckman reported mounting evidence that ADHD is a significant risk factor for smoking.^19^ Biederman et al. also found a link with nicotine and future substance use disorders; those who smoked were more likely to drink alcohol and use illicit drugs.^20^ Wender asserts emotional over-reactivity and mood instability are highly suggestive of adult ADHD, integral to its phenomenology.^21^ Many adults with ADHD experience mood instability and short fuse temper outbursts throughout a lifetime.^4^ Referring to all the categories of functional impairment, the pattern among these parents is similar to that seen in speciality centres where adult ADHD was diagnosed using structured psychiatric interviews.^5^

### Adapted JDQ driving constructs compared with ADHD symptoms

In this study, the parents showed a heterogeneous outcome in driving style, risk and behaviour suggesting impairment in executive functioning as an underlying latent construct. An increase in driving accidents occurs because of distraction, impulsivity and having an increased need for stimulation.^11^

Recent literature indicates an increased risk of motor vehicle collisions and other driving offences in young drivers with ADHD.^15^ This study has older drivers who still have problematic behaviour and driving styles not resolved with age, suggesting driving impairment can predict ADHD in absent or reduced ADHD symptoms.

### DSM-5 accordance

The results of this study regarding the WFIRS-S, JDQ part B, ASRS- V1.1 are in accordance with DSM-5; the parents could be provisionally diagnosed with unspecified ADHD (no collateral history) and/or ADHD specified otherwise (partial or insufficient symptoms) as valid diagnostic groups. This supports the DSM-5 criteria that is accounting for adult symptom clusters, including delayed presentation of functional impairment. Jerome and Segal suggest driving impairment should be included in screening tools in the future for ADHD.^15^

## Study limitations and directions for future research

The small study of 81 has lowered the power of the tests to detect statistically significant effects and theta would have shown up in a bigger sample. However, the limitations of the small subsample sizes were reduced and the overall representation of the parent samples were improved by using validated scales^14,15^ and non-parametric tests.

Research on driving skills on its own and ADHD is practically absent in South Africa. South Africa has one of the highest motor vehicle accident rates in the world.^22^ Our study had few valid drivers and the findings were alarming as older drivers showed impaired driving skills and behaviours. More studies are needed as ADHD and driving has medico-legal implications and applications for the general psychiatrist.

## Conclusion

This study contributes to the literature on ADHD and the parent/s living with an ADHD child. In keeping with other studies on ADHD, the findings suggest that there are a large number of undiagnosed adults with ADHD even in a South African context associated with significant impairment in functioning.

It is evident that the age of onset criteria for ADHD is less reliable and important than the persistence of symptoms and functional impairments of ADHD across the lifespan.

These findings suggested that a significant number of parents who screened negative for ADHD and were parenting an ADHD child, may have experienced parental stress or could suffer from undiagnosed ADHD with comorbidities, as reflected by reporting impairment in functioning.

This study reflected a strong association between poor child mental health (CADHD) and parent outcome. All mental health care workers should be cognisant of impaired clinical functioning in these vulnerable parents, which might indicate undiagnosed ADHD especially in those having a strong familial history of ADHD and parental stress. To achieve better clinical outcomes in these susceptible parent-child systems, parents should be promptly referred for further psychiatric assessment as well as parent management training.

Future development should include the integration of DSM-V and the JDQ to develop and validate new appropriate screening tools for adults to identify on-going life difficulties in these vulnerable adults for ADHD and poor functioning.^13^ Future research should embark on case controlled longitudinal studies including follow-up studies in treated cohorts.
